# Psychiatric disorders and aggression in the printed media: is there a link? a central European perspective

**DOI:** 10.1186/1471-244X-12-19

**Published:** 2012-03-12

**Authors:** Alexander Nawka, Tea Vukušić Rukavina, Lucie Nawková, Nikolina Jovanović, Ognjen Brborović, Jiří Raboch

**Affiliations:** 1 Department of Psychiatry, First Faculty of Medicine, Charles University in Prague, Prague, Czech Republic; 2 Andrija Štampar School of Public Health, Medical School, Zagreb University, Zagreb, Croatia; 3 Department of Psychiatry, University Hospital Centre, Medical School, Zagreb University, Zagreb, Croatia

## Abstract

**Background:**

A content analysis was used to describe the association between psychiatric disorders and aggression in the printed media in the Czech Republic and Slovakia.

**Methods:**

Articles were chosen from the most widely read daily newspapers and magazines in both countries during five one-week periods in 2007. A coding manual was developed and a content analysis was performed. Aggressive behavior was assessed by two separate categories - the role of the mentally ill person in the violent act (perpetrator/victim) and the type of aggressive act (homicide, suicide).

**Results:**

A total of 375 articles were analyzed. Main findings: 1) The proportion of articles depicting psychiatric disorders together with either self- or other-directed aggressive behavior is 31.2%; 2) Homicide was most frequently mentioned in the context of psychotic disorders and schizophrenia, while affective disorders were most frequently associated with both completed suicides and homicides; 3) Eating disorders and anxiety disorders were seldom associated with any kind of aggressive behavior, including self-harm; 4) The vast majority of articles presented mentally ill people as perpetrators, and these articles were more often coded as stigmatizing. 5) Articles with aggressive behavior mentioned on the cover are roughly as frequent as those with aggressive behavior in the later sections of the media (36.7% vs. 30.7%).

**Conclusions:**

The results are similar to the findings in countries with longer histories of consistent advocacy for improved depiction of mental illness in the media. However, we have shown that persons with mental illness are still over-portrayed as perpetrators of violent crimes, especially homicides.

## Background

Media are considered to be the public's primary source of information regarding mental health [1-3]. Over the past five decades, starting with Nunnally's studies in the 1960's, the media coverage of psychiatric disorders has repeatedly been the subject of empirical research. Mass media representations of people with psychiatric disorders, in particular those with substance abuse and schizophrenia, tend to emphasize violence, dangerousness, and criminality [4-6]. It has also been shown that attitudes of people who see a significant linkage between psychiatric disorders and violence are based, to a large extent, on the information presented in the media [7]. Such attitudes are then widely spread among the public, where they persist, become a part of the culture [8], and ultimately result in discrimination against people with psychiatric disorders [9].

Negative presentations of psychotic and affective disorders prevail in the printed media in United States, United Kingdom, and Canada; and the most frequent topics of these negative articles were violence and dangerousness of mentally ill persons toward other people [10-12]. Corrigan et al. reports that 39% of all articles covering topics of psychiatric disorders focused on dangerousness and criminal activities [13]. The crucial issue, however, is that individuals with psychiatric disorders who are under the influence of drugs or alcohol are *not *significantly more likely to commit violent crime than the general population [11,14], with the implication that media accounts are biased and stigmatizing. Empirical data are showing that even severe mental illness alone did not predict future violence, which was rather associated with historical (past violence, physical abuse), clinical (substance abuse), dispositional (age, sex, income) and contextual (recent divorce, unemployment) factors [15].

Moreover, in Italy, Carpiniello et al. found a significantly higher number of words, accompanying photos, and more stigmatizing language in reports dealing with deeds attributed to people with psychiatric disorders [16]. Additionally, these articles were also more often published on the cover of newspapers and magazines, which increases their potential to influence the readers [11,13].

### Situation in Czech republic and Slovakia

Because the Czech Republic (with a population of 10.2 million) and the Slovak Republic (with 5.4 million) [17] once comprised a single country, the history of psychiatry and mental health care issues is very similar, as is the socio-cultural background. During the communist era of 1948 - 1989, the one-party system with a centralized economy and substantial restrictions of democratic rights influenced all aspects of society. No civic movements or non-governmental organizations existed to provide advocacy, promotion, prevention, or rehabilitation. Only the main political, social, and administrative changes after the "Velvet Revolution" in November 1989 provided the basis for the creation of new mental health policies, which enabled attempts to address stigma [18].

There is scarce evidence on media coverage studies involving persons with psychiatric disorders in Central and Eastern Europe. In the Czech Republic, these issues were investigated only marginally as a part of the studies done in relation to the image of drug abuse and drug users [19,20]. Recently, our team finished a comprehensive study on the depiction of mental illness/health issues in the printed media in the Czech Republic, Slovakia, and Croatia [21].

The present study extends this report, with the goals of understanding (a) how often, in media accounts, aggressive behavior is linked to persons with psychiatric disorders; (b) which psychiatric disorders are depicted together with aggressive acts; (c) what type of aggressive acts are mostly portrayed in articles depicting psychiatric disorders, and (d) identification of the role of a person with psychiatric disorder in aggressive deeds (i.e., as perpetrator or victim).

## Methods

Our study sample comprised articles pertaining to the topic of psychiatric disorders chosen from the six most widely read printed newspapers and magazines in both countries. This represents more than 75% of all readers of all newspapers and more than 50% of all magazines [22]. Articles were retrieved by a media agency and were taken from five one-week periods throughout the year 2007. Articles from both countries were analyzed as a single pool, as the Czech Republic and Slovakia share similar socio-economical, political, and mental health policies. All parts of the newspapers were searched for key words (neutral terms, e.g., depression, dementia, as well as labeling terms, e.g., schizophrenic, alcoholic) covering all the major psychiatric disorders, including news, interviews, columns, and editorials.

We used content analysis [23] to study articles in which a person with a psychiatric disorder represented the relevant content obtained after establishing the keywords.

Out of all articles obtained after setting the keywords, we preformed relevance sampling of the articles in which the subject of psychiatric disorder represented the relevant content. The initial numbers of articles identified were 1424 in the Czech Republic and 900 in Slovakia. The initial articles' search revealed numerous articles that did not use keywords in association with persons with psychiatric disorders, for example: "*Depression *on the stadium after losing the game; New anti-corruption law caused *anxiety *in the parliament, *Alcoholic *beverages cannot be advertised"; etc. The selection of articles with psychiatric disorder as the relevant content defined our final sample. A total of 375 articles were identified for the further analysis; 203 in the Czech Republic and 172 in the Slovak Republic.

This sampling is a combination of *purposive sampling *[24] focusing on key media, using only the six most read print media, *stratified composite sampling*, randomly selecting units over a time period (stratification by weeks or days) which has been identified as the most accurate sampling method for analysing media publications [25], and then after stratification by weeks or days the third step included *relevance sampling *[26], sampling of relevant content from those media based on a keywords search and the association of the article with psychiatric disorders, either as the main subject of the article or a sideline to another story.

These articles were further analyzed according to the PICMIN instrument's initial version developed for the purpose of this study [27]. Its development was based on the theoretical framework of content analysis [23,28,29].

The PICMIN instrument's initial version is composed of descriptive and analytical categories. Descriptive categories were used for easy identification of separate items and for finding the links with analytical categories. Within the analytical ones, aggressive behavior was assessed by two separate subcategories. In the first, the role of the mentally ill person in the violent act was recorded (focusing on whether the person with psychiatric disorder was depicted as a perpetrator or a victim of violent acts). In the second, we identified the particular type of aggressive act (homicide, physical assault, aggression against objects, completed suicide, attempted suicide and self-harm). In assessing the global impression of the article, the items were evaluated according to the presence of stigmatizing/de-stigmatizing statements and coded as either negative, positive, mixed (both statements present), or neutral (none of the statements present). Each analytical category included a paragraph-long definition to facilitate coding [21,27].

Articles were coded as positive if the article: a) supported a positive picture of the mentally ill or psychiatric service by portraying it in a way that a mentally ill person is included in society, able to socially participate; b) presented examples of mental illness professionals, institutions or NGO's providing help to the mentally ill, their families and society; c) articles avoided reinforcing stereotypes of mental illnesses and were respectful of people's rights. Articles were coded as neutral if the article stated the facts in an objective way and did not give information which might sway the reader's perspective on mental illness. Articles were coded as negative if: a) mentally ill persons were portrayed as violent or dangerous; b) mentally ill persons were connected with criminal behaviour, endangering society; c) pejorative and colloquial terms were used and d) media presentations of mental illness promoted negative images and stereotypes. Articles were coded as mixed if both positive and negative impressions were found in their content.

To reflect the overall tone or global impression of the article related to stigma, six positive or negative themes pertaining to the topic of mental health/illness were defined "a priori". A list of themes was generated based on previous research [10,13,30-32], and through a consultation process with the project's mentors. Each coder was supplied with this list with themes expressed in a list of statements, such as: "Treatment is beneficial"; "People with mental illness can socially function in the community", "People with mental illness are usually violent/aggressive", etc. Coders could choose one or more, if appropriate, of the six "a priori" defined themes, or write their own conclusion with the main message of the article within the open-ended box in the on-line version of the instrument that was used for data entries. These positive/negative themes served as a basis for the coding of the "global impression of the article" [27].

Disorders that were included generally corresponded to classifications from the International Classification of Diseases [33] and were grouped in the following clusters: Organic disorders (F00 - F09), Substance abuse disorders (F10 - F19), Psychotic disorders (F20 - F29), Affective disorders (F30 - F39), Neurotic disorders (F40 - F48), Eating disorders (F50), "Other psychiatric disorders" (F51 - F99) and "Not related to any specific psychiatric disorder". "Other psychiatric disorders" stands for personality disorders, including antisocial personality disorder; child and adolescent disorders, including conduct disorder; mental retardation and sexual disorders.

All categories were defined a priori by the research team during several workshops and were used as a basis for consensual coding. Reliability of the coding among raters in both countries was assured by their uniform training and regular international meetings, in which they discussed possible differences in interpretation. Inter-rater reliability (IRR) was determined for the descriptive and analytical categories using the indices: *Average Pair-wise Percent Agreement *(APPA) *and Krippendorff's α *(alpha). APPA is a more liberal index, comprising a single comparison of the level of agreement among coders and ratings, whereas and *Krippendorff's α *(alpha), is a more conservative index of co-variation applicable to nominal and categorical data, which accounts for agreement expected by chance. *Krippendorff's α *(alpha) values ≥.60 were considered reliable; values ≥.75 indicated high reliability [34-36]. IRR was calculated with the ReCal ("Reliability Calculator"), an online utility that computes inter-coder reliability coefficients [37].

Descriptive statistics were used to present all obtained data. The differences of various frequencies of variables were determined using *χ*^2 ^tests. For cases in which the *χ*^2 ^test is not appropriate, the *p *value vas calculated using robust non-parametrical Monte Carlo test for independence of rows from columns. For each genuine table 99999999 random tables were made. Each random table has the same marginal totals as its genuine table. The random tables come from a population having independent rows and columns. A p value < .05 was considered statistically significant [38]. All statistical analyses except the Monte Carlo test were carried out with SAS 9.1 statistical software package

## Results

The APPA for categories "aggressive act" and "aggressive behavior" was more than 88% and Krippendorf's α was over .71 in both countries. APPA and Krippendorf's α values for the category "global impression of the article" (*de-stigmatizing, neutral, mixed or stigmatizing*) were below satisfactory reliability; hence the final rate for that category was made by consensus among all three raters per country.

One third of the analyzed articles (N = 117, 31.2%) reported that persons with psychiatric disorders were involved in some kind of aggressive behavior. A vast majority of these articles presented mentally ill individuals as perpetrators (N = 84, 71.8% articles). In 19 articles (16.2%), they were presented as victims of aggressive behavior; in 14 articles (11.9%) they were placed in the role of both victim and perpetrator at the same time. The rest of the analyzed articles (N = 258, 68.8%), contained no information that a person with psychiatric disorder was involved in aggressive activity.

Persons with psychotic disorders were most frequently presented as perpetrators (N = 24, 50.0%), whereas persons with organic disorders were more often presented as victims of aggressive behavior (N = 2, 12.5%). Eating disorders (N = 30, 93.8%) and anxiety disorders (N = 22, 91.7%) were most often presented without any mention of aggressive behavior (Table 1).

**Table 1:** Portrayals of persons with different psychiatric disorders according to their role in the aggressive behavior mentioned in the article, N (%)

Disorder mentioned	F0 N = 16	F1 N = 86	F2 N = 48	F3 N = 69	F4 N = 24	F50 N = 32	Other PD N = 30	NRSPD N = 127	Total N = 375
Perpetrator	3 (18.8)	14 (16.3)	24 (50.0)	10 (14.5)	0 (0)	0 (0)	6 (20.0)	33 (26.0)	84 (22.4)
Victim	2 (12.5)	2 (2.3)	3 (6.3)	4 (5.8)	2 (8.3)	2 (6.3)	2 (6.7)	6 (4.7)	19 (5.1)
Both	3 (18.8)	5 (5.8)	6 (12.5)	3 (4.4)	0 (0)	0 (0)	2 (6.7)	3 (2.4)	14 (3.7)
No aggressive behavior	8 (50.0)	65 (75.6)	15 (31.3)	52 (75.4)	22 (91.7)	30 (93.8)	20 (66.7)	85 (66.9)	258 (68.8)

Monte Carlo test, *p *< .0001

F0 stands for organic disorders, F1 stands for substance abuse disorders, F2 stands for psychotic disorders, F3 stands for affective disorders, F4 stands for neurotic disorders, F50 stands for eating disorders, "Other PD" stands for personality disorders, including antisocial personality disorder; child and adolescent disorders, including conduct disorder; mental retardation and sexual disorders; and NRSPD stands for Not related to any specific psychiatric disorders

Self-directed aggression behavior was reported in 19 (5.1%) articles mentioning completed suicide, 27 (7.2%) with attempted suicide, and in 8 (2.1%) articles self-harm was addressed. Completed suicide most often appeared in articles dealing with affective disorders (N = 9, 2.4%), whereas attempted suicide was mostly mentioned in "non related to any specific psychiatric disorder" articles (N = 18, 4.8%). Homicide cases were mentioned in 51 articles (13.6%) and various forms of physical assault toward other people in 48 (12.8%) articles. Aggression against objects was revealed in 22 (5.9%) articles. Although homicide was associated mostly with psychotic (N = 19, 5.1%) and affective disorders (N = 13, 3.5%), cases of physical assault were most frequently mentioned in articles dealing with subjects with psychotic (N = 15, 4.0%), and organic disorders (N = 4, 1.1%). Aggression against objects appeared mostly in articles coded as "others" (N = 4, 1.1%), which dealt with child and adolescent disorders and personality disorders.

Articles in which persons with psychiatric disorders were presented as perpetrators were more frequently coded as stigmatizing, while those in which they were presented as victims were more frequently coded as mixed. Articles without any aggressive behavior were on the other hand significantly more often coded as de-stigmatizing or neutral (Table 2).

**Table 2:** The global impression of the article and the role of person in the aggressive behavior mentioned, N (%)

Global impression	De-stigmatizing (N = 126)	Neutral (N = 38)	Mixed (N = 53)	Stigmatizing (N = 158)
Perpetrator	2 (1.6)	1 (2.6)	4 (7.6)	77 (48.7)
Victim	1 (0.8)	2 (5.3)	5 (9.4)	11 (7.0)
Both	3 (2.4)	1 (2.6)	3 (5.7)	7 (4.4)
No aggressive behavior	120 (95.2)	34 (89.5)	41 (77.4)	63 (39.9)

Monte Carlo test, *p *< .0001

The proportion of articles on the cover with aggressive behavior mentioned is similar to the later sections of the media. Type of media (newspapers vs. magazines) did not have any impact on the proportion of articles in regard to the aggressive behavior mentioned. The length of the article with and without aggressive behavior mentioned did not differ significantly, either (Table 3).

**Table 3:** Various characteristics of the articles with or without aggressive behavior mentioned, N (%)

		Aggressive behavior mentioned	No aggressive behavior mentioned
Position of the article*	On cover (N = 30)	11 (36.7)	19 (63.3)
	Later sections (N = 345)	106 (30.7)	239 (69.3)
Type of printed media**	Newspapers (N = 340)	106 (31.2)	234 (68.8)
	Magazines (N = 35)	11 (31.4)	24 (68.6)
Words count**^¥^**		371.6	381.7

*Χ^2 ^= 0.4540 df = 1, p = .5005 **Χ^2 ^= 0.0009 df = 1, *p *= .9755 **^¥ ^***t*-test, p = .8243

### Comparison with other countries

A similar prevalence (31%) of articles depicting psychiatric disorder together with aggressive behavior against self or others was observed in the United States 39% [13] and in Serbia 32% [39], but higher rates were found in the UK 46% [11] and in New Zealand 61% [10]. Only Australian researchers found that stories related to psychiatric disorders in the context of crime were relatively uncommon in the printed media (6%) [40].

### Organic disorders

Although organic disorders (mostly dementia) were mentioned in only 4% of the articles, the fact that half of these cases were mentioned in the context of some kind of aggressive behavior should not be overlooked. It has been suggested that subjects with dementia often become agitated and violent at home or in psychiatric facilities [41], but rarely are they engaged in well-planned and pre-meditated killings [42]. Looking at this problem from a different perspective, Gerkin and colleagues analyzed characteristics of male criminals with organic brain syndrome and found that those with an early onset of criminal activity (by age 18) show a more global, persistent, and stable pattern of offending than those with a late onset [43]. Negative stereotyping of organic disorders may pose a significant threat to society's perception of old age, thus increasing the likelihood of organic disorders joining psychotic disorders on the top of the "most dangerous disorders".

### Psychotic disorders

Not surprisingly, schizophrenia was most frequently mentioned in the context of homicide (40% of the articles). While epidemiological investigations are consistently showing that the proportions of persons with schizophrenia who commit crimes vary from one study to another, the elevations in risk among those with schizophrenia when compared to the general population remains similar [44]. In a systematic review using meta-analysis, Large and colleagues found that a pooled proportion of 6.5% of all homicide offenders had a diagnosis of schizophrenia [45]. Given that the lifetime prevalence of schizophrenia is estimated to be between 0.5% and 1%, there is a disproportionate number of homicide offenders with schizophrenia. Fazel et al. identified 20 studies that compared the risk of violence in people with schizophrenia and the risk of violence in the general population. In conclusion, although people with schizophrenia were nearly 20 times more likely to have committed murder than people in the general population, only one in 300 people with schizophrenia had killed someone, a similar risk to that seen in people with substance abuse [46].

### Affective disorders

Both suicides and suicide attempts were most frequently reported in the context of affective disorders, which reflects the observation that out of all psychiatric disorders, depression and bipolar disorder poses the highest risk for suicide [47,48]. Furthermore, a significant proportion of articles reported on subjects with affective disorders who committed homicide. This finding contradicts a study by Rove and colleagues indicating that depression was rarely associated with violence and the focus was generally on self-harm [49]. However, subjects with bipolar affective disorder and substance abuse co-morbidity are reported to commit more violent crimes than the general population [42]. Nevertheless, it is important to emphasize that suicide and homicide are both extremely complex phenomena that depend on many factors, not just the diagnosis.

### Neurotic and eating disorders

Not unexpectedly, eating disorders together with neurotic disorders received the best coverage in terms of connection with dangerousness. None of the 56 articles dealing with neurotic or eating disorders portrayed these patients as criminal offenders. Of particular interest is the fact that among 32 articles that mentioned eating disorders, none reported on self harm even though the lifetime rate of self-injurious behavior occurrence in person with eating disorders is as high as 34% [50].

### Substance abuse disorders and "other psychiatric disorders"

Substance abuse and antisocial personality disorders are commonly reported as co morbidities which are significantly contributing to the increase incidence of violence in people with severe mental illness [15,51]. Likewise persons with severe mental illness who have a history of conduct disorder by mid-adolescence are at increased risk for aggressive behaviour and violent crime [42]. Even though the substance abuse disorders received the widest coverage among the main diagnostic clusters, interestingly in terms of revealing the patient as a perpetrator it did not by far reach the high prevalence of psychotic disorders (16% vs. 50%), in contrary, the vast majority of such articles (76%) did not mention violent crime at all.

Conduct Disorder in Childhood and Antisocial Personality Disorder in adulthood were not treated as separate diagnosis because of extremely low frequency of endorsement. This is a notable finding, as the evidence is showing that the individuals with antisocial personality disorder have very often criminal history [52], e.g. prisoners are about ten times more likely to have antisocial personality disorder, than the general population [53]. When analyzing the whole cluster "other psychiatric disorders" that included these conditions, distinct link with violence commitments has been traced. This is however mirrored in the printed media less dramatically than one would anticipate based on the evidence.

### Psychiatric patient: Criminal or victim?

A vast majority of articles in our study presented mentally ill people as perpetrators. Corrigan et al. found that only 4% of the articles portrayed mentally ill people as victims, which is very close to our finding of 5% [13]. A high perpetrator/victim ratio may falsely suggest that mentally ill individuals are more likely to be the aggressive initiators of violence rather than victims of aggressive behavior, even though victimization is more common than aggressive behavior among these individuals [42]. Similar studies in other countries reported that stories related to aggressive behavior often ended up in the front sections of newspaper, making them more visible to readers [13,40,54], but this was not the case in our sample, as those articles were almost equally distributed on the cover as in the later sections.

### Joint action of mental health professionals and journalists needed

Although there is an association of homicide with specific psychiatric disorders, particularly in respect to phases of illness in schizophrenia [51], antisocial personality disorder, and/or drug or alcohol abuse, the dominance of dangerousness and criminality depictions in the media of mental illness is overstated. Psychiatric disorders in general do increase the risk of homicidal violence by two-fold in men and six-fold in women [55], but our findings reveal disproportionate depictions of violence and aggression in all main psychiatric diagnostic clusters except for neurotic and eating disorders.

Still, our results may be viewed as promising, as they are relatively similar to the findings in countries with a much longer history of consistent activities of advocacy groups that are trying to change the information the public is given about mental illness. In our case, special efforts to de-stigmatize mental illness in the media should be directed toward the psychotic disorders, especially schizophrenia and organic disorders, as their prevalence in recent decades has been rising steeply. A possible future study therefore might be a review of articles depicting homicides during the same period of time as in presented study. This will enable us to determine what proportion of all homicides committed and presented in the print media mentioned psychiatric disorder, especially psychotic and organic ones.

### Study limitations

Several limitations of this study need to be considered. The authors used five one-week periods as the time frame, so some stories may have been overrepresented in the final sample. Secondly, the sample of the articles could be limited by the keywords. Most importantly, our analyses are based on a raters' interpretation of the articles and on the final consensus agreement of all raters in each country. Some level of unreliability among country ratings might be possible, but still, the languages are very similar and even a few inconsistencies among countries were discussed. In comparison with other studies, where different coding schemes were utilized, direct comparisons may suffer from some imprecision. As in almost all other studies in this field, only written materials are analyzed, so important visual information, such as photos, were omitted in the coding process.

## Conclusion

Even in the current era of global internet, printed media still present a powerful channel through which information can be transferred to society. Regarding depictions of mental illness, newspapers and magazines have become social structures for perpetuating stigma [13]. Therefore, there is still an imminent need to start organized activities like joint education workshops for journalists and mental health professionals in order to re-shape the negative stereotypes and attenuate the stigmatizing potential surrounding mental illnesses. We encourage journalists and other media-related professionals to work on more comprehensive depictions of persons with mental illness, which will reflect a more accurate reality of their lives. This will not only benefit those suffering so far from the negative stereotyping of mental illness, but also society as a whole by enhancing the overall quality of media representations.

## Competing interests

The authors declare that they have no competing interests.

## Authors' contributions

AN, TVR and LN participated in the design of the study. AN and LN were involved in the data collection. NJ and JR helped draft the manuscript and partially participated in the design of the study. OB performed the statistical analysis. All authors read and approved the final manuscript.

## Pre-publication history

The pre-publication history for this paper can be accessed here:

http://www.biomedcentral.com/1471-244X/12/19/prepub
